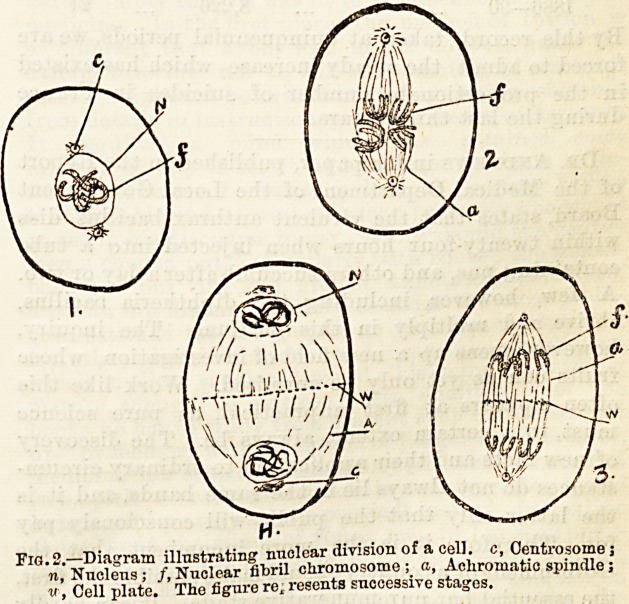# Modern Biology

**Published:** 1893-12-02

**Authors:** 


### Modern Biology.

### IV.—CELL DIVISION.

It has already been stated that the essential parts which go to make up a cell, are constituted by the cytoplasm and the nucleus within it. It is possible, however, that this expression may have to become less simple, owing to discoveries now being made in the field of cellular histology. The facts upon which this suggestion rests are briefly as follows:

In many cells, especially in those which ultimately form the reproductive elements, there is seen, in a

Fig. 1.—*A*, Ascaris megalocephala (ovum); *B*, The same cell preparing to divide; *a*, Archoplasm; *b*, Centrosome; *f*, Nuclear fibril; *n*, Nucleus. *p*, Cytoplasm.

position more or less contiguous with the nucleus, a mass of protoplasm which differs from the surrounding cytoplasm in its density, as well as in the rapidity and tenacity with which it reacts to stains. This body, to which the name of archoplasm has been given, is often of a definite form, and frequently contains a clear space within it, in which again is situated a granule, termed the centrosome. (Fig. 1.) In some cases the archoplasm has been seen without an obvious centrosome, in others the centrosome is clear, while an archoplasm cannot, at least as yet, be distinguished. But these structures, although of common occurrence in reproductive cells, have not as yet been seen in all the body-cells. It is possible their presence may ultimately prove to be universal, but at least there is no evidence as yet to show it. They certainly, however, occur occasionally, as for example in the dividing leucocytes and pigment cells of some animals, and probably in certain body-cells of plants. When present, they are seen to exercise a remarkable influence upon the process of nuclear division in every case.

When a nucleus is about to divide, and this always takes place before cell-division, extremely complicated processes become at once apparent. The fibrillar structure, alluded to in the last article, becomes thicker, and the chromatin substance embedded in it, denser; finally, the convoluted thread, or threads, split up while still within the nucleus, into thick segments which may assume various shapes, from that of spheres to that of loops; the particular form exhibited is, however, characteristic for the particular species of plant or animal. What is more singular still, is the fact that the number of segments is also constant for the species, and that a definite difference exists between the number of the segments in the reproductive cells, and that of the body cells, at least in many investigated cases. This is of such a kind, that there are present twice as many of the segments in the body-cell nuclei as in the reproductive-cell nuclei. Shortly after the formation of the definite number of fibrillar segments, the nucleolus commonly disappears, though what precisely becomes of it is not at present known. The nuclear membrane, at the same time, disappears also, so that the segments of the nuclear fibril, or chromosomes, as they are termed, come to be exposed to the surrounding cytoplasm. But changes have also, in the meantime, been going on in this; the centrosome, when present, has become doubled; the two structures more apart, and the protoplasm (or archoplasm) between them assumes the form of threads which form a barrel or spindle-shaped sheaf of fibres having a centrosome at each pole. This structure is known as the achromatic spindle. It is sometimes formed away from the nucleus, but more often the centrosomes move round this body and occupy diametrically opposite positions. Then, as they separate and the spindle is formed, they pull the nucleus out into an elliptical form. If, on the other hand, the spindle is formed at a distance from the nucleus, so that this is not at first included, sheaves of threads diverge from the main portion and, so to speak, catch up the nucleus, so that the final result is the same. When the nuclear wall disappears the threads of the achromatic spindle become attached to the chromosomes, which by this time have congregated to form a plate-like arrangement in a position occupying the equatorial plane of the spindle. And now another remarkable occurrence is seen, the preparatory steps of which have been visibly going on for some time previously. Each chromosome splits into exactly two halves by a longitudinal division, so that there are now twice as many chromosomes at the equator as before. Next, the chromosomes begin

Fig. 2.—Diagram illustrating nuclear division of a cell. *c*, Centrosome; *n*, Nucleus; *f*, Nuclear fibril chromosome; *a*, Achromatic spindle; *v*, Cell plate. The figure represents successive stages.

to move away from the equator, along the achromatic spindle, towards its two poles. The process, however, standing in a definite relation with the duplication just mentioned; and this relation is of such a nature that if we imagine each original chromosome to split into a blue and a red half, all the blue daughter-segments go in one direction, and all the red ones in the other; and in this way it is probable that a qualitative and quantitative similarity between the two young daughter nuclei is secured. The achromatic spindle now extends across the whole space originally occupied by the mother nucleus, and eventually the daughter nuclei are formed from the two bundles of retreating chromosomes in such a manner that the same stages are gone through, only in a reverse order, which occurred as preparatory to the original division. The chromosomes lengthen, form the convoluted thread, and finally the young nuclei surround themselves with their proper walls, while nucleoli appear within them. Meanwhile, at the equator of the spindle thickenings occur on the threads, and these fuse to form a "cell plate." In animals this is not often easy to follow, but in plant cells, on the other hand, it is very obvious, and it ultimately cuts the original mother cell into two daughter cells. The plate primarily consists of nitrogen-containing substances, but, at least in plants, it is eventually replaced by a cellulose skin. There are many other subsidiary phenomena connected with cell division which will be discussed later, but the main point now to be insisted on is that the cell division is preceded by nuclear division, and that this is of an extremely complicated character. J. B. F.

## Figures and Tables

**Fig. 1. f1:**
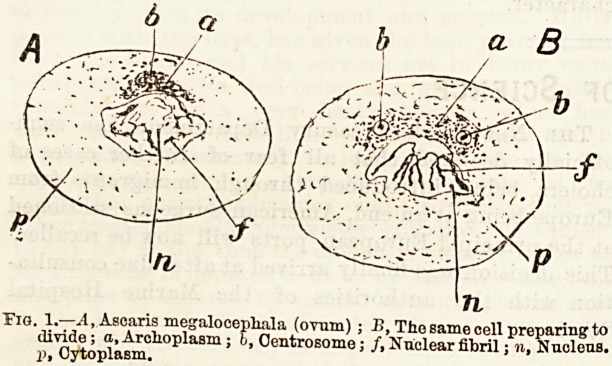


**Fig. 2. f2:**